# Fungal profile, levels of aflatoxin M1, exposure, and the risk characterization of local cheese ‘*wagashi*’ consumed in the Ho Municipality, Volta Region, Ghana

**DOI:** 10.1016/j.toxrep.2024.01.009

**Published:** 2024-01-17

**Authors:** Nii Korley Kortei, Valentina Sylvia Gillette, Michael Wiafe-Kwagyan, Leslie Owusu Ansah, Vincent Kyei-Baffour, George Tawia Odamtten

**Affiliations:** a Department of Nutrition and Dietetics, School of Allied Health Sciences, University of Health and Allied Sciences, PMB 31, Ho, Ghana; b Department of Sports Nutrition, School of Sports and Exercise Medicine, University of Health and Allied Sciences, PMB 31, Ho, Ghana; c Department of Plant and Environmental Biology, College of Basic and Applied Sciences, University of Ghana, P. O. Box LG 55, Legon, Ghana; d Department of Food Laboratory, Food and Drugs Authority, P.O. Box CT 2783, Cantonments, Accra, Ghana; e Food Chemistry and Nutrition Research Division, Council for Scientific and Industrial Research, Food Research Institute, P. O. Box M20, Accra, Ghana

**Keywords:** Aflatoxin M1, Cancer, Hepatocellular carcinoma, *wagashi*, Milk products, Fungi species

## Abstract

*Wagashi* is a West African type cottage cheese locally prepared from cow milk. *Wagashi* like other milk products, is prone to microbial contamination, particularly by fungi. Many of these fungal species produce mycotoxins which are of serious public health concern. This work aimed to update the mycoflora profile and determine the concentrations of aflatoxin M1 and its health risk characterization due to the consumption of *wagashi.* Culturing the *wagashi* on mycological media (Oxytetracycline Glucose Yeast Extract OGYE, Dichloran Rose Bengal Chloramphenicol DRBC) caused a de-novo growth of the quiescent spores at 28–30 °C for 5–7 days. The analysis of AFM1 levels in the samples was done using High-Performance Liquid Chromatography connected to a Fluorescence detector (HPLC-FLD). The exposure and risk assessment to the AFMI levels were determined using deterministic models prescribed by the European Food Safety Authority (EFSA). The fungal counts ranged between 2.36–4.30 log10 CFU/g. In total, thirteen (13) fungal species from eight (8) genera were isolated from all *wagashi* samples. They are; *Fusarium oxysporum, Aspergillus flavus, Aspergillus niger, Fusarium verticillioides, Penicillium digitatum, Trichoderma harzianum, Aspergillus terreus, Rhodotorula mucilaginosa, Rhizopus stolonifer, Aspergillus fumigatus, Yeast sp., Mucor racemosus* and *Fusarium oligosporum* belonging to the genera *Fusarium, Aspergillus, Penicillium, Trichoderma, Rhodotorula, Rhizopus, Yeast,* and *Mucor*. The AFM1 observed in the *wagashi* samples' analysis was low, ranging from 0.00 (Not Detected) ± 0.00 − 0.06 ± 0.002 µg/Kg. Risk assessments of AFM1 using deterministic models produced outcomes that ranged between 5.92 × 10^−3^- 0.14 ng/kg bw/day, 1.42 –44.35, 0–0.0323 ng aflatoxins/kg bw/day, and 1.51 × 10^−3^ − 9.69 × 10^−4^ cases/100,000 person/yr for estimated daily intake (EDI), margin of exposure (MOE), average potency, and cancer risks, respectively, for the age categories investigated. Fungal counts were interpreted as medium to high. It was also established that the consumption of *wagashi* may pose adverse health effects on all age categories in the selected zones of the study since all calculated MOE values were less than 100,000.

## Highlights

• The majority of the ‘wagashi’ samples were infected with toxicogenic fungi.
• Fungal counts of samples exceeded limits set by the International Microbiological Specifications of Foods.
• Risk assessment revealed an exposure to cancer and was of public health concern.

## Graphical Abstract

**Figure:**
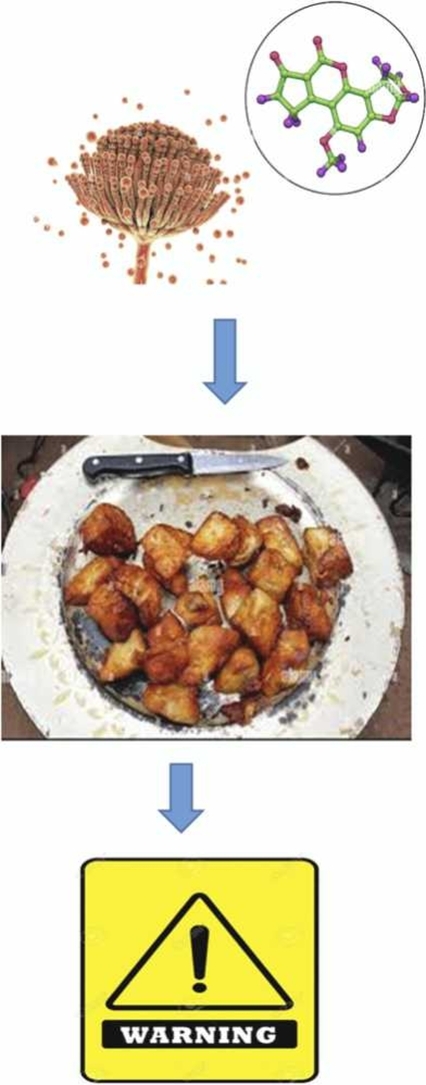

## 1 Background

Raw milk provides all the necessary nutrients and conditions for the growth of many fungal species. The quality of the nutrient and ability to support fungal growth is influenced by the animal’s physiological state, aeroclimatic, and breeding conditions [77]; Negash, 2018). Raw milk from animal sources is obtained from cows, goats, sheep, buffaloes, camels, and other lactating ruminants. Among these, cow milk constitutes eighty-three percent of the world’s milk production, and is more affordable [56]. Cow milk is also the most common source of raw milk within typical Ghanaian Hausa and Fulani communities. Unfortunately, it is found to be laden with the highest fungal diversity as compared to ewe and goat milk [33]. Because of its availability and affordability, several by-products are manufactured from it for human consumption; among these is the locally-made cheese called *wagashi*.

Cheese is a dairy product with significant therapeutic and nutritional benefits. The soft unripened cheese known as "*wagashi*" is popular throughout West Africa, particularly in Ghana, Benin, and Nigeria (where it is known as "*wagashi*," "wara," or "warankashi") [12]. Milk production in Ghana is carried out predominantly by pastoralist nomadic Fulani, who are mostly engaged in moving their free-range feeding cattle from one location to another. "Wagashi" is a product that is made mostly by Fulani women to preserve extra raw milk for a short time. In the Northern and Volta areas of Ghana, *wagashi* is primarily consumed at home or sold in large quantities on the market [66].

The growth of stored and harvested foods containing fungi species mainly from the genera *Aspergillus, Penicillium,* and *Fusarium* results in the production of toxic secondary metabolites of fungi called mycotoxins.

Mycotoxins are heterogenous groups of toxic secondary metabolites produced by toxigenic fungi before harvest and in storage that contaminate a wide range of cereals, nuts, cocoa, fresh and vegetables, fruits, etc., and their derived processed products with over 600 having been characterized. The mycotoxins frequently contaminating human and animals foods are aflatoxins (B1, B2, G1, G2, M1), ochratoxin A, fumonisins (B1, B2, G2), zearalenone, trichothecenes (dexynivalenol, T2 toxins, HT2 toxins, nivanelol) penicillic acid, patulin is considered to be significant [10], [55], [61], [132]. The availability of safe food is a *sine qua none* for the well-being of people and the development of national economies. Unfortunately, the low quality and safety of foods in Africa significantly impact human and animal health and are a major constraint to growers who need access to more remunerating markets [84]. Some factors that are a threat to food quality include poor physical quality, chemical contamination, and bacterial and mycotoxin contamination [18]. According to the Food and Agriculture Organisation, FAO, a quarter of all food crops are contaminated by mycotoxins [38]; Felicia Wu, 2007).

Aflatoxins (AF) are the most occurring of all mycotoxins in both human and animal foods [128]. Aflatoxins occur in 6 forms grouped as B1, B2, G1, G2, M1, and M2; all are potentially toxic, carcinogenic, teratogenic, and mutagenic agents [3], [19], [30], [112]. Aflatoxin B1 (AFB1) has carcinogenic, hepatotoxic, teratogenic as well as neurotoxic and neuro-immunotoxic effects [115] in action and a positive correlation has been established between the consumption of aflatoxin-contaminated food and the incidence of liver cancer in South East Africa and some Africa populations [25], [27]; McGlynn, Petrick, & El-Serag, 2021; [96]; H. C. [130] and are also anti-nutritional contaminants in many food commodities [127]. Aflatoxins pose the greatest risk to health in tropical Africa due to their widespread prevalence in foods. Indeed mycotoxin exposure is believed to contribute to more than 40% of the global disease burden [127].

Aflatoxins are di-furanocoumarin derivatives produced by the polyketide pathway by some strains of Aspergillus namely; *A. flavus, A. parasiticus, A. bombycis, A. ochraceous, A. nomius* and *A. pseudotamarii*, of all these aflatoxins-producing strains *A. flavus* is found to be the most common contaminant in agricultural produce [20], [98], [99], [109] including maize, groundnut, dried fruits, meat, milk and milk products [89].

*A. flavus* NRRL5906 in foods produces AFB1, AFB2, AFG1, and AFG2 concurrently [100], [99], [97] while other strains produce either only AFB’s or AFG’s. the International Agency for Research on Cancer [27], [82] (IARC, 1993) has classified AFB1 as a group 1 carcinogen often encountered in crops like maize, groundnut, dried fruits, meat, milk, and milk products. The same Agency IARC, classifies AFM1 in milk products as Carcinogen Group 2B.

Aflatoxin M1 (AFM1) is the main hydroxylate metabolite derived from aflatoxin B1 (AFB1) in the liver due to cytochrome enzymes and is often detected in milk and dairy products (MDPs) [40].

AFM1 contamination of milk and milk products globally is well established and has been reported in many countries including Ghana [62], [66], [71], [72]. It is excreted in milk in the mammary glands of both human and lactating animals [39], [40]. AFB1 is reported to have a significant association with AFM1 so it is possible to predict the outcome of AFM1 with knowledge of AFB1 [87]. Studies in recent times have been directed at reducing the bioavailability of AFB1 to reduce the levels of AFM1 in lactating dairy cows [87]. Approximately 0.3 – 6.2% of AFB1 is converted into metabolized AFM1 and excreted in variations, the milking process, and environmental conditions. It is known that AFM1 is about 10 times lower in its carcinogenic potential than AFB1 (Category Group 1). AFM1 is classified as Carcinogenic Group 2B by the IARC [82]; IARC 1993). However, AFM1 can still be a health hazard to humans, particularly children, considering their high milk consumption, lower body weight, high metabolic rate, and incomplete development of excretory organs [83]. Indeed, [66], reported that consumption of *wagashi* a soft unripened cheese in Ghana posed adverse health effects on all age categories in the selected regions of study because all calculated Margin of Exposure (MOE) values were less than 100,000. They concluded that contamination of *wagashi* with AFM1 should be a serious public health concern and as such should be considered a high priority for Ghana’s risk management actions. Interestingly, *wagashi* is also consumed in several parts of West Africa, mainly Nigeria where it is called *wara* or *warankashi* and Benin Republic (*woagachie*) [12], [66].

The presence of AFM1 in milk and dairy products is therefore an important health issue globally but developing countries are at a greater risk. The toxic effect of consuming aflatoxins in its minutest amount does have health-altering effects [107]. Consequently, many countries are implementing strict regulation of levels of aflatoxins in foods consumed by their populace. The aflatoxins limit usually set by countries in Europe is EFSA = 0.05 ug/kg and in other African countries e.g. Ghana (GSA = 0.5 ug/kg) to regulate the permissible levels to safeguard consumers from hepatocellular carcinoma (HCC) which is known to be the fifth most frequently occurring cancer in the world [48], [75]. Owing to this, the European Union (EU) has a stricter standard level for total aflatoxin not exceeding 4 ug/kg for direct consumption of any product [15]. In the United States, the maximum acceptable limit for AF is 20 ug/kg [129].

In addition to setting regulatory limits for mycotoxins, it is also worthwhile to conduct a health risk assessment on the populace due to dietary exposure. Risk evaluation is now widely accepted as the ideal means to assess possible links between hazards to the food chain and actual risks to human health (EFSA, 2008; Meeting & Organization, 2002).

Cheese which is a milk product with high nutritional and medicinal value is manufactured from raw milk from animal sources obtained from cows, goats, sheep, buffaloes, camels, and other lactating ruminants. Among these, cow milk constitutes eighty-three (83%) of the world's milk production and is more affordable [56], [88]. An unripened cheese product called *wagashi* is consumed in several parts of West Africa including Nigeria (under the name *wara* or *warankashi*) and Benin (under the name *wagashi*). In Ghana this cheese product is called *wagashi* [12]. Nomadic Fulani who are pastoralists are mainly involved in the rearing of cattle in the West Africa region and take along their grazing cattle from one place to another. In the process, the Fulani women use the lactation product in milk production in Ghana. *Wagashi* is indeed a processed product obtained when there is excess raw milk as a way of processing the excess milk for the short term mainly by Fulani women. In the Northern and Volta Region of Ghana, *wagashi* is primarily consumed at home or widely sold in the open market in some regions of Ghana. It is either eaten as a whole meal or consumed in combination with some foods.

According to Delavenne et al. [33], cow milk contains the highest fungal species load (population) as compared to other types of milk consumed by humans. This leaves much to be desired. The presence of fungi in such food may predispose the milk samples to contamination by mycotoxins released as secondary metabolites of the resident fungi.

*Wagashi* (which is a cow milk product) is amenable to aflatoxin M (AFM1) contamination as AFM1 is the main hydroxylated metabolite of aflatoxin B1 (AFB1) in the liver due to cytochrome enzymes and has been detected in milk and dairy products (MDP’s) in Ghana [66], [71], [72] and elsewhere [40]. It is well known that mycotoxin contamination in dairy cow nutrition is mainly caused by the resident mycoflora of harvested corn and silage [23]. When a dairy cow consumes approximately 40 ug/kg of AFB1, daily produced as a result of feeding on contaminated silage and corn by fungi (especially *Aspergillus flavus and A parasiticus*) [21], [78] the milk it produces contains 0.05 ug/kg (highest level) of AFM1 (Meeting & Organization, 2002). According to the AFB1 contamination level in feeds about 0.30 to 6.2% of AFB1 consumed by dairy animals is chemically modified into AFM1 [16].

Neither storage nor processing of the milk into cheese can eliminate AFM1 from the milk and its products. AFM1 has been detected even in pasteurized milk, UHT milk, milk powder, milk formulae, yogurt, feta cheese, white cheese and traditional cheese, ice cream, and butter [26]. Cheese is a principal source of aflatoxins among milk products owing to the correlation of AFM1 with casein fraction in milk, which is primarily concentrated in cheese [86], [92]. Current data in the pertinent literature show that the concentration of AFM1 is about thrice greater in various soft cheeses and around fivefold greater in hard cheese than in milk from which cheese is manufactured [63], [86]. Campagnollo, et al., [26] and Kortei & Annan, [66] showed that AFM1 contamination in cheese may be exacerbated by poor processing methods of the milk. Additionally, ingestion of aflatoxins worsens the risk of hepatocellular carcinoma (HCC) in man which is reported to be the fifth most frequently occurring cancer in the world [79], [131]. Epidemiological and animal studies have shown that hepatitis B virus (HBV) and AFM1 surge the likelihood of occurrence of HCC in people with hepatitis B surface antigen-positive (HBsAG+) by 3 – 5 fold [94]. Furthermore, Neuveut, Wei, & Buendia, [93] showed that pre-existing liver disease caused by HBV infection compromises the ability of hepatocytes to debilitate carcinogens such as aflatoxins thus increasing the chance of HCC.

There are limited references to work done on the prevalence of fungi in milk and milk products in Ghana [66], [72]. This sequel study provides novel information on the prevalence of toxigenic resident fungi and especially AFM1 in *wagashi*, (a local cheese) eaten in Ho Municipality of the Volta Region of Ghana. Risk assessment evaluation is provided to assess possible links between hazards to the food chain and actual risks to human health at all ages across the board.

## 2 Methodology

### 2.1 Study area and site

**Fig. 1:**
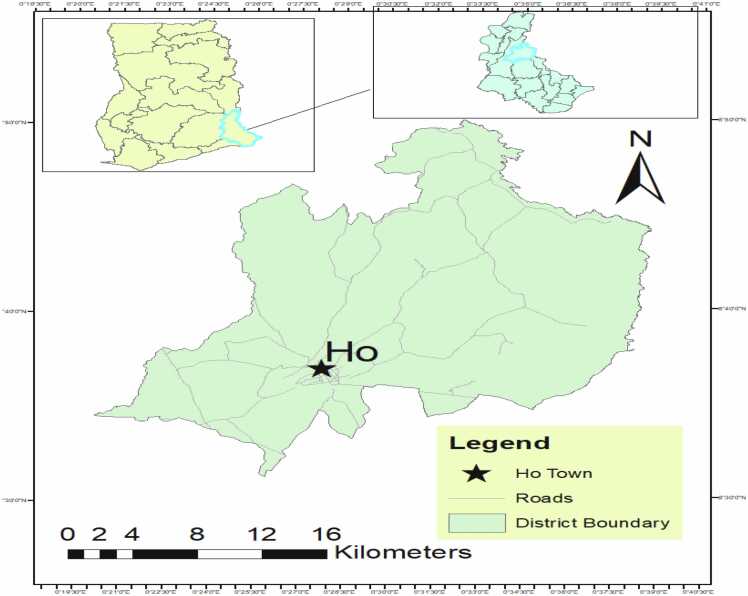
Map of Ghana showing Ho in the Volta Region.

This experimental work on this paper was conducted at the Microbiological Laboratory of the School of Allied Health Sciences at the University of Health and Allied Sciences. Ho, Volta Region within the Ho Municipality. Ho Municipal is the administrative capital and economic hub of the Volta Region of Ghana and forms part of the 25 municipalities and districts of the region. It consists of 772 communities and a land size of 2660 sq. according to the records of the Ghana Statistical Service (GSS, 2014). Fig. 1.

### 2.2 Sampling of *Wagashi*

A total of 54 samples but composited to 18 different samples were obtained in this survey. Within the indicated darkened Ho municipality, the sites of sampling for this study were categorized into six (6) zones from the reference point of Ahoe, which is the most central location within the Municipality. Three (3) samples were purchased from each of the zones. Table 3.1 shows these different zones A - F.

### 2.3 Sampling method

A purposive sampling method was used. Fried *wagashi* samples were purchased from different *‘waakye’* (a meal of boiled rice and beans with pepper sauce) sellers in the various indicated sites (Table 1) within the Ho Municipality from July 2021 to August 2021. A total of eighteen (18) samples in triplicates of the traditional local fried soft cheese samples were bought from six (6) differently zoned locations in Ho municipality where the Fulanis (Nomads) and *‘waakye’* sellers are located in Ho. The ‘*wagashi*’ samples were collected and stored in sterile specimen bags (Nasco, USA) and kept in an Ice Chest (Thermos, 7750, China) with cold packs to maintain the temperature of 10 °C under aseptic conditions. The samples were transported to the laboratory where they were stored at low temperatures until they were ready for analysis [69].

**Table 1:** Communities from which *wagashi* samples were obtained within the Ho municipality.

Zone Category	Communities Sampled
A	Power House Nogora Heve
B	Trafalgar Godokpe Dave
C	Bankoe Civic Center Ahoe
D	Ho main market Fiave Deme
E	Ho (market top) Dabra (2 different sellers)
F	Akoefe (2 different sellers) Ho (market down)

### 2.4 Sample categorization

Samples were labeled appropriately as they were collected. The labelling format used was; food name *wagashi* (W) followed by zone letters (A, B, C, D, E, F) and sample number (1−3) such that label ranged from WA1 to WF1 to WF3 (a total of 18). (Table 1).

## 3 Sample size determination

Cochrane formula for the determination of smaller sample populations.

n = no.

1 + [(no-1) +N], where n = sample size (new adjusted sample size).

no= sample size.

N = population size.

n = 40.

1 + [(40−1)+ 44 = 18.

### 3.1 Microbiological Analysis

#### 3.1.1 Fungal plating and incubation

One gram (1 g) of each sample was transferred into 9 mL of sterile distilled water. The samples were soaked overnight. All samples were weighed using an electronic balance (OHAUS®, Germany) with a readability of 0.01 g. Each stock solution was serially diluted in 9 mL of peptone (0.1%) water in tenfold increments from stock 10^0^ to 10^−3^. One milliliter (1 mL) of each serial dilution was plated in either Dichloran Rose Bengal Chloramphenicol (DRBC) (Oxoid CM727) or Oxytetracycline Glucose Yeast Extract (OGYE) (Oxoid CM727). Agar media plates were prepared according to the manufacturer’s instructions and incubated at 25 °C for 5–7 days as outlined by Kortei, Asiedu, Annan, Deku, & Boakye, [68] and Odamtten, Nartey, Wiafe-Kwagyan, Anyebuno, & Kyei-Baffour, [101].

#### 3.1.2 Fungal Enumeration and Identification

Enumeration was carried out using a colony counter. Fungal counts were recorded after 7 days and later conventionally transformed into a logarithmic scale log_10_ CFU/g [101].

(1) $$\mathrm{CFU}/g=\frac{\left(\mathrm{no}.\mathrm{of}\;\mathrm{colonies}\;x\;\mathrm{reciprocal}\;\mathrm{of}\;\mathrm{dilution}\;\mathrm{factor}\right)}{\mathrm{volume}\;\mathrm{of}\;\mathrm{cutlure}\;\mathrm{plate}}$$

Percentage occurrence of fungal species was calculated using the formula;

(2) $$\mathrm{Percentage}\;\left(\%\right)\mathrm{occurrence}\;\mathrm{of}\;\mathrm{fungal}\;\mathrm{species}=\frac{\mathrm{number}\;\mathrm{of}\;\mathrm{fungal}\;\mathrm{species}}{\mathrm{total}\;\mathrm{number}\;\mathrm{of}\;\mathrm{fungi}\;\mathrm{isolated}}\;x100$$

#### 3.1.3 Identification

Moulds that appeared were identified by their culture and morphological characteristics using Standard Identification Manuals [116].

According to the International Commission for Microbiology Specifications for Food (ICMSF) (ICMSF, 1996) shown in Table 2, the quality of the *wagashi* samples from Zones A-F was below standard (marginal - potentially injurious) and unfit for human consumption.

**Table 2:** Guidance on the interpretation of results for specific foodborne pathogens of foods in general (CFU/g).

Hazard	Result (CFU/g)	Interpretation	Likely cause
Fungi	< 10^2^ or 2 log_10_	Satisfactory	
	10^2^ to < 10^4^ or 2-4 log_10_	Marginal/borderline	Process controls not fully achieved or possible raw material contamination
	> 10^4^ or 4 log_10_	Unsatisfactory (potentially injurious to health and/or unfit for human consumption)	Inadequate time and temperature control during cooling and subsequent storage allowing spores to germinate and multiply

Source: International Commission for Microbiologcal Specfications for Food (ICMSF, 1996)

### 3.2 Mycotoxin analysis

#### 3.2.1 Preparation of samples

The samples were warmed at 37 °C in a water bath with constant temperature before they were blended to increase their surface area and centrifuged at 2000 g to separate fat layers before filtration and then filtered. The prepared test portion of 50 mL was transferred into a syringe barrel attached to the AFM1 immunoaffinity column and passed at a slow steady flow rate of 1–2 mL/min. The columns were then washed with 20 mL deionized water and the air was passed through the columns to dryness. AFM1 was eluted with 4 mL pure acetonitrile by allowing it to be in contact with the column for about 60 s. The eluate liquid was evaporated to dryness using a gentle stream of nitrogen. The residue was then dissolved in 500 µl of mobile phase and filtered using a membrane filter before being injected into the High-Pressure Liquid Chromatography column for quantity estimation of AFM1.

#### 3.2.2 Chemicals and standards preference

The AFM1 standard was supplied by Sigma-Aldrich (St. Louis, MO, USA). All solvents to be used for the preparation of the mobile phase were HPLC grade and were purchased from Merck (Darmstadt, Germany). All homogenized mixtures and eluates were filtered through Whatman no. 4 and 0.45 mm membrane filters, respectively (Whatman plc, Maidstone, UK). De-ionized water was obtained from a Millipore Elix Essential purification system (Bedford, MA, USA). EASI-extracted AFM1 immunoaffinity columns (stored at 4 °C) were supplied by R-Biopharm, Rhone Limited, and used for SPE and cleanup.

#### 3.2.3 Preparation of standard solutions

A stock solution (0.1 μg/mL) was prepared from a standard solution of AFM1 (0.993 μg/mL in acetonitrile) and stored with care in the freezer. A working stock solution of 0.01 μg/mL was diluted step by step with the combined solution (acetonitrile/ water, 75/25, v/v) to prepare a sequence of working solutions that were stored in vials below 4 ºC for the calibration curve. Calibration solutions of 0.02 μg/kg, 0.04 μg/kg, 0.06 μg/kg, 0.08 μg/kg, and 0.10 μg/kg were used. Samples with AFM1 amount above the calibration range were diluted and dilution factors were applied for quantification.

#### 3.2.4 Instrumentation

Agilent high-performance liquid chromatography system (HPLC 1260 Infinity Series, OpenLab Software, X-bridge column) (250 mm × 4.6 mm, i.d., 5 µm, USA) with a quaternary pump and fluorescence detection were used for AFM1 quantification analysis and were carried out according to the method of EN ISO 14501:2007 [121]. Data acquisition and quantification were done using Chem station (Open Lab edition). The Agilent HPLC was set at an excitation wavelength of 360 nm and an emission wavelength of 440 nm and the column compartment (HPLC Column: TC-C18 (2), 170, 5 µm, 4,6 × 250 mm; with, a pore size of 170, particle size of 5.0 µm, inner diameter of 4.6 mm, length of 250 mm and carbon load of 12%). Temperature was regulated at 35 °C. The mobile phase was a mixture of water and acetonitrile at ratios of 25:75 (v/v), respectively, and an isocratic delivery mode was employed at a flow rate of 0.8 mL min^−1^ with an injection volume of 50 µl.

#### 3.2.5 Validation

The HPLC-FLD method was validated according to the guidelines prescribed by the European Commission Decision 657/2002/EC for confirmatory analysis methods and the tested parameters were: linearity, limit of detection (LOD), limit of quantification (LOQ), accuracy, precision, and selectivity. The linearity was assessed by constructing five-point solvent-matched calibrations in triplicate for AFM1 standard solutions in the concentration range of 0.05 to 0.8 mg/L. Calibration curves were drawn by plotting the peak area against AFM1 concentration, and linearity was evaluated by linear regression analysis expressed as coefficient of determination (r^2^).

The precision of the method was estimated in terms of % RSD of three identical extractions of milk samples spiked with AFM1 at the same as well as at three different spiking levels. Method selectivity was evaluated by analyzing AFM1 known negative milk matrix and reagent blank to determine any interference from endogenous substances around the retention time of the target analyte.

### 3.3 Human risk assessment of exposure to AFM1 via consumption of cheese ‘*wagashi*’

#### 3.3.1 Exposure estimation

Estimated Daily Intake (EDI) was calculated by using the mean quantities of aflatoxins derived from the ‘*wagashi*’ milk samples, the number of samples consumed daily, and the average body weight. The EDI for mean aflatoxin was according to the following formula (3) below and expressed in μg/kg of body weight/day (μg/kg bw/day) [29], [34];.

(3) EDI = *daily* int*ake* (food) X mean level of Aflatoxins

Average bodyweight.

The daily intake of milk products in Ghana [66], [104] is approximately 0.0137 kg/day (5.0 kg/year).

The average body weight of the different age categories was calculated according to the EFSA Panel on Dietetic Products & Allergies, [35] and their corresponding estimated average weights in Ghana were used in this study as follows: Infants - 2.9 kg (2.5–3.2 kg) [4], [74], Toddler - 9.8 kg (7–12.6 kg) [1], [47], Children - 26 kg (24–28 kg) [24], [106], Adolescents - 46.25 kg (38.5–54 kg) (Afrifa–Anane et al., 2015), Adults - 60.7 kg [125].

#### 3.3.2 Population risk characterization for aflatoxins (Hazard Index, HI)

Genotoxic and carcinogenic compounds such as aflatoxins have their risk assessment calculated based on the Margin of Exposure (MOEs) approach which MOEs are estimated by dividing the Benchmark dose lower limit (BMDL) for aflatoxins – 0.2 ng/kg bw/day by toxin exposure as spelt out by [7], [49]; Meeting & Organization, 2001) expressed in Eq. (4).

(4) MOE= Benchmark dose lower limit

EDI (Exposure).

For AFM1, TDI was 0.2 ng/kg/day, which was obtained by dividing TD50 (threshold dose per BW) with a variability factor of 5000. A public health alarm was raised in instances where MOEs are less than 100,000 [7]; Meeting & Organization, 2001).

#### 3.3.3 Estimated liver cancer risk due to consumption of Wagashi samples

The ingestion of aflatoxins can be linked to the onset of liver cancer [41], [57], [126]. Therefore, liver cancer risk estimation for Ghanaian adult consumers was calculated for aflatoxins [7], [67], [70], [103]. This involved estimating the population cancer risk per 100,000, which is a product of the EDI value and the average hepatocellular carcinoma (HCC) potency figure from individual potencies of Hepatitis B surface antigen (HBsAg) (HBsAg-positive and HBsAg-negative groups).

The JECFA [59] estimated potency values for AFM1 which corresponded to 0.3 cancers/year/100,000 population ng/kg bw/day (uncertainty range: 0.05–0.5) in HBsAg-positive individuals and 0.01 cancers/year/100,000 population/ng/kg bw/day (uncertainty range: 0.002–0.03) in HBsAg-negative individuals [119] was adopted for this calculation. Moreover, the average HBsAg+ prevalence rate of 7.74% (adult-8.36%, 14.3%-adolescents, 0.55%-children) for Ghana [2], [102] was adopted and 92.26% (100 – 7.74%) was extrapolated for HBsAg-negative groups. Hence, the average potency for cancer in Ghana was estimated as follows according to Eq. (5) as prescribed by Shephard, [119], Kortei, Annan, Kyei-Baffour, et al., [72] and Adetunji, et al., [7].

(5) Average potency = [0·03 x HBsAg – negative individuals in Ghana] + [0·01 x HBsAg- positive individuals/prevalence rate in Ghana]

= (0.3 × 0.077) + (0.01 × 0.9226).

= 0.0323 cancers per year per 100,000 population per ng aflatoxins/kg bw/day.

Thus, the Cancer risk (cancers per year per 100,000 population per ng aflatoxin/kg bw/day) was estimated using the following formula in Eq. (6) [7]; [57]:

Thus, the population risk was estimated using the following formula in Eq. (6):

(6) Cancer Risk = Exposure (EDI) × Average potency

### 3.4 Statistical analysis

The aflatoxin concentrations were calculated using regression analysis from the curves generated from the standards of aflatoxin M1 with Excel for Microsoft Windows (version 16). One-way ANOVA was used to compare the means that were obtained and a 5% level of significance (p < 0.05) was used with a 95% confidence interval. The statistical results were summarized as means, standard deviation, and SPSS 22 (Chicago, USA) were used in the analysis of data. Deterministic risk assessment model calculations for aflatoxins, dietary exposure (Estimated Dietary Intake), MOE values, average potency, and cancer risk were calculated.

## 4 Results

### 4.1 Fungal populations

Figure 4.1 shows results of the mean fungal populations which were calculated as log_10_CFU/g. The mean fungal population obtained on DRBC agar for *wagashi* samples obtained from zone A ranged from 2.36 to 3.32 log_10_ CFU/g. Zone A samples were not statistically different in their fungal counts (p = 0.49). In zone B, the value ranged from 2.75 to 3.56 log_10_ CFU/g. There was no statistically significant difference (p = 0.52) between the samples from this zone. Zone C recorded a range of 2.99 to 3.78 log_10_ CFU/g. There was also no significant difference (p = 0.53) between zone C samples. Zone D fungal counts ranged from 3.06 to 3.54 log_10_ CFU/g and were statistically not different (P > 0.05) (p = 0.84). In Zone E, the fungal population ranged from 3.8 to 4.04 log_10_ CFU/g while Zone F had fungal counts ranging from 3.79 to 4.3 log_10_ CFU/g. There was therefore no statistically significant difference (p = 0.96) (p = 0.84) between samples in zones E and F respectively.

The results of the mean fungal populations obtained on OGYE agar media from the *wagashi* samples are presented in Figure 4.2 Results obtained here showed the same trend as on DRBC. Zone A samples had mean fungal population ranging from 4.07 to 4.2 log_10_ CFU/g, and there was no statistically significant difference (p = 0.99). In zone B, fungal population ranged from 3.88 to 4.09 log_10_ CFU/g; there was no statistically significant difference (p = 0.96). Zone C fungal populations in *wagashi* ranged from 3.91 to 4.1 log_10_ CFU/g and again there was no statistically significant difference (p = 0.98). Mean fungal population counts in zone D ranged from 3.93 to 4.06 log_10_ CFU/g; there was no statistically significant difference (p = 0.98). In zone E, values ranged from 3.97 to 4.12 log_10_ CFU/g and there was no statistically significant difference (p = 0.98). Finally, in zone F samples, fungal population ranged from 3.96 to 4.14 log_10_ CFU/g and there was no statistically significant difference (p = 0.97) in the range of values obtained for each replicate at the respective sites.

**Fig. 2:**
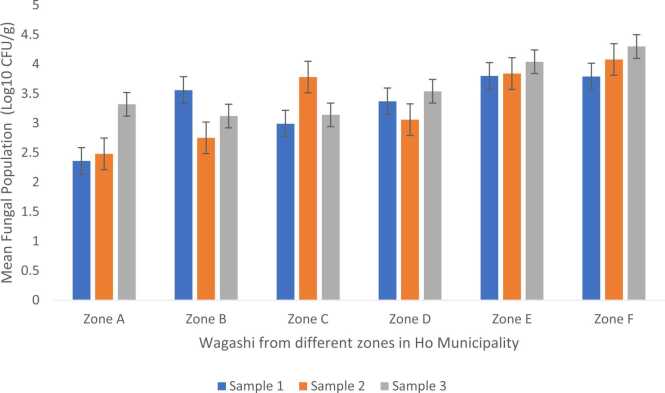
Mean fungal population in *wagashi* samples from 6 different zones within Ho municipality isolated with DRBC.

Results obtained on DRBC agar media (Fig. 2) indicate that Zone A had the lowest mean fungal count of 2.36 log_10_ CFU/g and the highest count was found in Zone F (4.3 log_10_ CFU/g).

Growth on OGYE agar media of the *wagashi* samples presented the lowest mean fungal population from Zone B (3.88 log_10_ CFU/g) and the highest from zone A (4.2 log_10_ CFU/g) differing significantly (P ≤ 0.05) from what was obtained on DRBC. The type of media therefore influenced the growth of the fungi isolated.

### 4.2 Fungal profile percentage (%) occurrence

Thirteen (13) different fungal species belonging to eight (8) genera were isolated from all *wagashi* samples from zones A – F: (Table 3, Table 4). They were; *Aspergillus flavus, Aspergillus niger, Asperfillus terreus, Aspergillus fumigatus, Fusarium oligosporium, Fusarium oxysporum, Fusarium verticilliodes, Mucor racemosus*, *Penicillium digitatum, Rhizopus stolonifer, Rhodotorula mucilaginosa, Trichoderma harziarum, Yeasts,* and belonging to the genera *Aspergillus, Fusarium, Mucor*, *Penicillium, Rhizopus, Rhodotorula, Trichoderma, Yeast sp*.

**Table 3:** Percentage (%) occurrence and the total number of different fungal species in *wagashi* isolated on DRBC agar at the indicated sites (locations) after 7 days at 28 – 32 °C.

Fungal species	Percentage (%) Occurrence on DRBC agar																	
	WA1	WA2	WA3	WB1	WB2	WB3	WC1	WC2	WC3	WD1	WD2	WD3	WE1	WE2	WE3	WF1	WF2	WF3
*Aspergillus niger*	23	14	10	40	80	15	33.6	8	40	10	23	8	-	38	-	-	37	-
*A. flavus*	10	7	-	-	6	-	8	-	5	5	-	-	7.4	8	-	7	9	-
*A, fumigatus*	-	-	-	4	-	7	8	10	7.5	-	9.4	7	-	7	-	-	-	-
*Fusarium verticilliodes*	-	15	-	-	-	42	-	-	-	-	-	-	-	-	-	-	-	-
*Aspergillus terreus*	5	-	-	-	-	-	-	2.5	5	-	-	-	-	-	-	-	-	-
*Fusarium oxysporum*	34	40	-	-	-	-	21	50	18	10	30	-	50	-	-	38	-	-
*Mucor racemosus*	-	-	-	-	-	-	-	-	-	-	4.6	-	-	-	-	-	3	-
*Penicillium digitatum*	20	24	-	38	-	22.5	26.4	20	20	45	-	19	-	-	-	-	-	-
*Rhizopus stolonifer*	-	-	-	3	-	-	-	2	4.5	-	-	6	-	-	-	-	-	-
*Rhodotorula mucilaginosa*	-	-	-	7	7	13.5	3	7.5	-	30	3	60	-	-	-	-	-	-
*Trichoderma harzianum*	8	-	-	-	7	-	-	-	-	-	15	-	12.6	-	-	-	11	-
*Yeasts*	-	-	90	8	-	-	-	-	-	-	15	-	30	47	100	55	40	100
Total	6	5	2	6	4	5	6	7	7	5	7	5	4	4	1	3	5	1

WA – WF (*Wagashi* Zones A-F*)*1-3: Replicates

**Table 4:** Percentage (%) occurrence and the total number of different fungal species in *wagashi* isolated on OGYE Agar at the indicated sites (locations) after 7days at 28 – 32 °C.

Fungal Species	Percentage (%) Occurrence on OGYE Agar																	
	WA1	WA2	WA3	WB1	WB2	WB3	WC1	WC2	WC3	WD1	WD2	WD3	WE1	WE2	WE3	WF1	WF2	WF3
*Aspergillus niger*	1.7			38.3	1.7	26.7	18.3	15		1.7	1.7							
*A. flavus*	-				18.3				5		1.7		5					3
*A. fumigatus*	-			1	5		1.7	1.7								5		
*A. terreus*	-	3.3																
*Fusarium oxysporum*	0.7	3.3	4.7	8.3	13.3	6.7	6.3	16.7		1.7	1.7		23.3					6.7
*F. verticillioides*	-												1.7	20				1.7
*F. oligosporum*	8.3			3.3	3.3	5		3.3			1.7							
*Penicillium digitatum*	3.3			5	5	8.3	11											
*Rhodotorulla sp*	-	32.7	3.3	0.7	1.7	10	4.7	1.7	16.7	1.7	6.7	3.3				5	80	16.7
*Mucor sp*	1.67	0.3	1						0.7	11.7	3.3	3.3	11.7		1.7			4
*Rhizopus stolonifer*	-		1					1.7					1.7		1.7			1.3
*Trichoderma harzianum*	-			1.7		3.3	1.3		5	1.7		6.7						1.6
*Yeasts*	84.3	60.3	90	41.7	51.7	40	56.7	60	72.7	81.7	83.3	86.7	56.7	80	96.7	90	20	65
Total	6	5	5	8	8	7	7	7	5	6	7	4	6	2	3	3	2	8

WA – WF (*Wagashi* Zones A-F*)*

1-3: Replicates

Table 3, Table 4 summarise the results of the isolation of fungal species from the two test media DRBC and OGYE. All the listed fungi could be isolated on the two media except for *Fusarium oligosporum* and *Mucor* which were absent on OGYE (Table 4). The percentage occurrence and total number of fungal species at each of the zones A – F were unique (Table 3; Table 4). *A. terreus* was the least encountered (WA1, WC2, WF2 on DRBC and WC2 on OGYE) whilst *yeast* predominated at all the sampling zones and sites ranging from 40 – 100% occurrence. Indeed at WE3 and WF3, only *yeast* was isolated on DRBC and 40 – 96.7% on OGYE, followed by *Fusarium verticilliodes*, *Rhizopus stolonifer,* and *Trichoderma harzianum* (Table 3, Table 4).

Some potentially toxigenic fungi species *A. niger, A. flavus, A. fumigatus, A. terreus, P. digitatum, and Rhodotorula mucilaginosa* were isolated at various times and locations (Table 3, Table 4, Table 7).

**Table 7:** Summary of the occurrence of fungal species resident in the *wagashi* cheese sampled from zones A -F in the Ho Municipality.

Fungal Species	Isolation Medium	
	**OGYE**	**DRBC**
*Aspergillus niger*	**+**	**+**
*A. flavus*	**+**	**+**
*A. terreus*	**+**	**+**
*A. fumigatus*	**+**	**+**
*Fusarium verticillioides*	**+**	
*F. oxysporium*	**+**	**-**
*F. oligosporum*	**+**	**+**
*Mucor sp*	**+**	**-**
*Penicillium digitatum*	**+**	**+**
*Rhizopus stolonifer*	**+**	**+**
*Rhodotorula mucilaginosa*	**+**	**+**
*Trichoderma harzianum*	**+**	**+**
*Yeast species*	**+**	**+**
**TOTAL**	**13**	**11**

**Plate 1:**
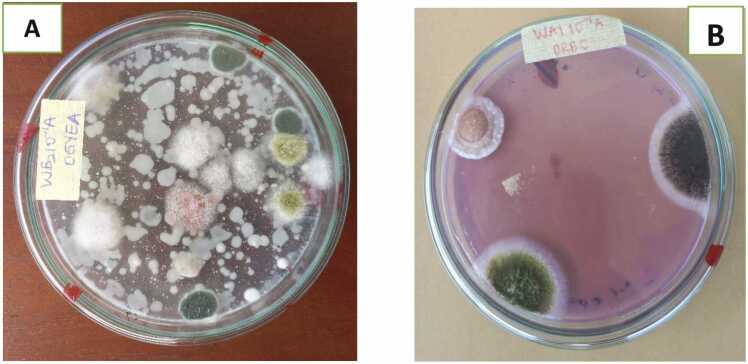
Plates showing macroscopic view of fungal species in wagashi A) Aspergillus flavus, Fusarium oxysporum, Penicillium digitatum, Yeasts spp**.** on OGYE agar B) Aspergillus flavus, A. niger growing on DRBC agar.

**Plate 2:**
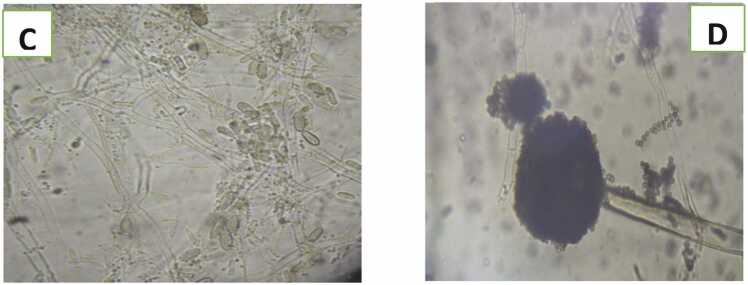
Microscopic view of C) Fusarium oxysporum (x400) and D) Aspergillus niger (x400).

Plate 1, Plate 2 show the gross cultural and microscopic morphologies of some of the fungi encountered.

### 4.3 pH of *Wagashi* samples

The pH of all 18 samples of *wagashi* is summarized in Table 5. The three replicate samples of each site A – F did not differ significantly (P ≥ 0.05) in pH (Table 5). However, there was a significant statistical difference (P ≤ 0.05) between pH recorded in *wagashi* from sampling sites A, B, C, D, and F.

**Table 5:** Pooled data of the total list of fungi isolated from *wagashi* purchased from different sites under laboratory conditions ERH 75–85% and 28–32 °C for 7 days.

*Aspergillus flavus* Link^WA1, WA2, WB1, WB2, WB3, WC1, WC2, WC3, WD1, WD2, WE1, WE2, WF1, WF2, WF3^
*Aspergillus fumigatus* Fresen^WB1, WB2, WB3, WC1, WC2, WC3, WD2, WD3, WE2, WF1^
*Aspergillus niger* Van Tiegher^WA1, WA2, WA3, WB1, WB2, WB3, WC1, WC2, WC3, WD1, WD2, WD3, WE2, WF2, WF3^
*Aspergillus terreus* Thom^WA1, WA2, WC2, WC3^
*Fusarium verticilliodes* (Sacc.) Nirenberg^WA2, WB3, WE1, WE2, WF3^
*Fusarium oligosporum* Saito^WA1, WB1, WB2, WB3, WC2, WD2^
*Fusarium oxysporum* Sehldt^WA1, WA2, WA3, WB1, WB2, WB3, WC1, WC2, WC3, WD1, WD2, WE1, WF1, WF3^
*Mucor sp*^WA1, WA2, WA3, WC3, WD1, WD2, WD3, WE1, WE3, WF2, WF3^
*Penicillium digitatum* Dierckx^WA1, WA2, WB1, WB2, WB3, WC1, WC2, WC3, WD1, WD3^
*Rhodotorula mucilaginosa*^WA2, WA3, WB1, WB2, WB3, WC1, WC2, WC3, WD1, WD2, WD3, WF1, WF2, WF3^
*Rhizopus stolonifer* (Ehrenb) Vuill.^WA3, WC2, WC3, WD3, WE1, WE3, WF3^
*Trichoderma harzianum* Rifai^WA1, WB2, WD2, WE1^
*Yeasts*^WA1, WA2, WA3, WB1, WB2, WB3, WC1, WC2, WC3, WD1, WD2, WD3, WE1, WE2, WE3, WF1, WF2, WF3^

Note- Superscripts indicate treatments in which fungi species occur

### 4.4 pH

The pH readings of all eighteen (18) *wagashi* samples are presented in Table 6. The pH recorded ranged from pH 5.60 to 6.83. There was no statistically significant difference (p > 0.05) among zone E samples. However, there was a statistically significant difference (p < 0.05) among zone A, B, C, D, and F samples.

**Table 6:** pH readings of *wagashi* samples from the indicated sampling sites.

Sample	pH	Mean ± standard deviation
WA1	5.99 6.02 6.03	6.01±0.02
WA2	6.16 6.18 6.18	6.17±0.01
WA3	5.92 5.93 5.94	5.93±0.01
WB1	6.52 6.56 6.58	6.55±0.03
WB2	6.80 6.84 6.84	6.83±0.02
WB3	6.24 6.23 6.24	6.24±0.01
WC1	6.02 6.05 6.07	6.05±0.03
WC2	6.03 6.04 6.05	6.04±0.01
WC3	5.60 5.60 5.61	5.60±0.01

WD2	6.17 6.21 6.22	6.20±0.03
WD3	6.30 6.33 6.34	6.32±0.02
WE1	6.25 6.28 6.29	6.27±0.02
WE2	6.28 6.30 6.31	6.30±0.02
WE3	6.25 6.26 6.28	6.26±0.02
WF1	6.01 6.00 6.00	6.00±0.01
WF2	5.68 5.69 5.69	5.69±0.01
WF3	6.35 6.39 6.41	6.38±0.03

Table 6 (cont’d) pH reading of *wagashi* samples from the indicated sample sites

### 4.5 Concentrations of the AFM1 in *wagashi* samples from different locations

**Fig. 4:**
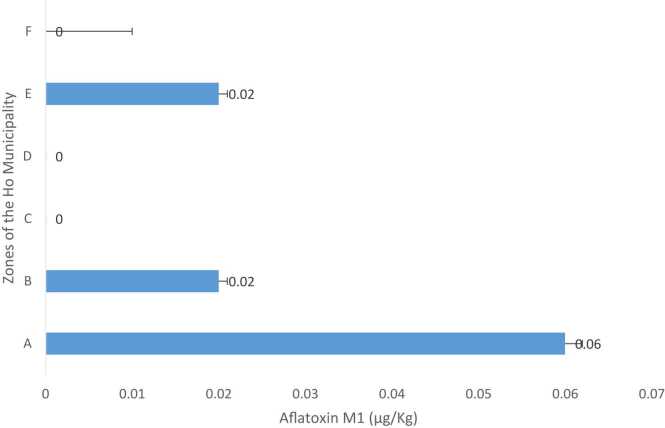
Mean AFM1 concentrations in *wagashi* obtained from the various zones of the Ho municipality *Note*- N.D- Not Detected.

The AFM1 concentrations obtained in the *wagashi* samples of the various zones of the Ho municipality were 0.0592 ± 0.002, 0.02 ± 0.001, 0.00(not detected) ± 0.00, 0.00(N.D) ± 0.00, 0.02 ± 0.001, and 0.00(N.D) ± 0.000 µg/Kg for zones A, B, C, D, E, and F respectively (Fig. 4). The concentrations of AFM1 analyzed were not statistically significant (P > 0.05). Concentration in zone A was significantly (P < 0.05) greater than all the other zones investigated. (Fig. 4).

### 4.6 Risk Assessment

The risk assessment for the consumption of *wagashi* by the different age groups (infants, toddlers, children, adolescents, and adults) is presented in Table 8. The estimated Daily Intake (EDI), Margin of Exposure (MOE) values, and cancer risk values were non-existent in zones C, D, and F because AFM1 could not be detected in the consumed *wagashi* (Table 8). On the other EDI of consumed AFM1 in *wagashi* from zone A varied from 0.141 ug/kg/bw/day (infants); 0.04 ug/kg/bw/day (toddlers); 0.03 ug/kg/bw/day (children); 0.018 ug/kg/bw/day (adolescent) to 0.014 ug/kg/bw/day (adults) (Table 8).

**Table 8:** Risk assessment for AFM1 via consumption of *wagashi* by different age groups.

Zone		Av. Body weight (kg)	EDI (ng/kg bw/day)	MOE	Cancer Risk Cases/100,000 person/yr)
A	Infants (0-11months)	2.9	0.141	1.42	4.55 × 10^−3^
	Toddlers (12-35months)	9.8	0.04	5.00	1.292 × 10^−3^
	Children (36months-10years)	26	0.03	6.67	9.69 × 10^−4^
	Adolescents (11-17years)	46.25	0.018	11.11	5.814 × 10^−4^
	Adults (18-64years)	60.7	0.014	14.24	4.52 × 10^-−4^
B	Infants (0-11months)	2.9	0.047	4.26	1.51 × 10^−3^
	Toddlers (12-35months)	9.8	0.014	14.29	4.52 × 10^−4^
	Children (36months-10years)	26	0.011	18.18	3.55 × 10^−4^
	Adolescents (11-17years)	46.25	5.92 × 10-3	33.78	1.91 × 10^−4^
	Adults (18-64years)	60.7	4.51 × 10-3	44.35	1.46 × 10^−4^
C	Infants (0-11months)	2.9	-	-	-
	Toddlers (12-35months)	9.8	-	-	-
	Children (36months-10years)	26	-	-	-
	Adolescents (11-17years)	46.3	-	-	^-^
	Adults (18-64years)	60.7	-	-	^-^
D	Infants (0-11months)	2.9	-	-	-
	Toddlers (12-35months)	9.8	-	-	-
	Children (36months-10years)	26	-	-	-
	Adolescents (11-17years)	46.3	-	-	^-^
	Adults (18-64years)	60.7	-	-	^-^

E	Infants (0-11months)	2.9	0.047	4.26	1.51 × 10^−3^
	Toddlers (12-35months)	9.8	0.014	14.29	4.52 × 10^−4^
	Children (36months-10years)	26	0.011	18.18	3.55 × 10^−4^
	Adolescents (11-17years)	46.3	5.92 × 10^−3^	33.78	1.91 × 10^−4^
	Adults (18-64years)	60.7	4.51 × 10^−3^	44.35	1.46 × 10^−4^
F	Infants (0-11months)	2.9	-	-	-
	Toddlers (12-35months)	9.8	-	-	-
	Children (36months-10years)	26	-	-	^-^
	Adolescents (11-17years)	46.3	-	-	^-^
	Adults (18-64years)	60.7			

Margin of Exposure-MOE

Means of aflatoxins M1- Zone *A*- 0.06 ± 0.002, Zone *B*-0.02 ± 0.001, Zone *C*- 0.00 ± 0.00, Zone *D*- 0.000 ± 0.00, Zone *E*-0.02 ± 0.001 µg/kg, Zone *F*- 0.00 ± 0.000 µg/kg

Daily intake of *wagashi* for infants was halved (0.5 ×0.0137 kg)

Average potency of aflatoxin= 0.0323

Average Body weights were obtained from the different ranges referenced by the authors

1 µg= 1000 ng

The Margin of Exposure (MOE) values for zone A are as follows: 1.42 (infants), 5.00 (toddlers), 6.67 (children), 11.11 (adolescents), and 14.24 (adults). (Table 8).

The estimated Daily Intake of AFM1 in zone B was different. They were 0.047 ug/kg/bw/day (infants), 0.014 ug/kg/bw/day (toddlers), 0.011 ug/kg/bw/day (children), 0.00592 ug/kg/bw/day (adolescent) and 0.00451 ug/kg/bw/day (adult). (Table 8). The corresponding MOE values were 4.26 (infants), 14.26 (toddlers), 18.18 (children), 33.78 (adolescents), and 44.35 (adults). The average potency of the aflatoxins was 0.0323 ug/kg/day aflatoxin and produced in zone A cancer risks of 4.55 × 0^−3^ (infants), 1.293 × 10^−3^ (toddlers), 9.69 × 10^−4^ (children), 5.814 × 10^−4^ (adolescents) and 4.52 × 10^−4^ (adults). (Table 8).

The Estimated Daily Intake (EDI) values of AFM1 in the *wagashi* obtained from zone E were as follows: 0.047 ug/kg/bw/day (infants), 0.014 ug/kg/bw/day (toddlers), 0.011 ug/kg/bw/day (children), 5.92 × 10^−3^ ug/kg/bw/day (adolescent), 4.57 × 10^−3^ ug/kg/bw/day (adults). The MOE values of AFM1 in zone E were 4.26 (infants), 14.29 (toddlers), 18.18 (children), 37.7 (adolescents), and 44.35 (adults). Cancer risks recorded were 1.51 × 10^−3^, 4.52 × 10^−4^, 3.55 × 10^−4^, 1.91 × 10 ^−4,^ and 1.46 × 10^−4^ respectively according to the age categories. In zones C, D, and F there no EDI, MOE, or cancer risk values (because no AFM1 was detected). However, the average potency of the AFM1 was the same for the zones.

### 4.7 Discussion

The raw milk used in the manufacture of *wagashi* is a vital source of micro- and macronutrients which is used to feed young mammals (Dror & Allen, 2011). Milk, because of its rich nutrient composition is highly susceptible to contamination originating from the animal, the environment, the source of feed for the animals, or the milk handlers. It could become a high-risk product that provides optimal conditions for the proliferation and survival of microorganisms. The presence of fungi in such food may predispose the milk samples to contamination by mycotoxin released as a secondary metabolite of the resident fungi.

In this present study, the mycological profile of the *wagashi* samples from six (6) zones in the Ho Municipality was determined. They belonged to the eight (8) genera (*Aspergillus, Fusarium, Penicillium, Trichoderma, Rhizopus, Rhodotorula, Mucor,* and *Yeasts*). *Aspergillus* species (*A. flavus, A. niger, A. fumigatus, A. terreus*) predominated followed by *Fusarium* (*F. oxysporium, F. oligosporum, F. verticillioides*) and single species of *Penicllium* (*P. digitatum*), *Rhizopus* (*R. stolonifer*), *Trichoderma* (*T. harzianum*), *Mucor sp, Rhodotorula* (*R. mucilaginosa*) and *yeasts*. (Table 5). As expected the occurrence of the fungal species at each sampling site was unique and considerable. However, *yeasts* predominated at all the sampling sites and *A. terreus* was the least encountered (Table 3, Table 4). The type of medium used in isolation influenced the type of fungi encountered and was reflected in the total fungal population and percentage of species encountered on OGYE (13) and DRBC (11) (Table 3, Table 4). For example, at WE3 and WF3 only yeasts were isolated on OGYE followed *by F. vertillioides, R. stolonifer* and *T. harzianum*.

**Fig. 3:**
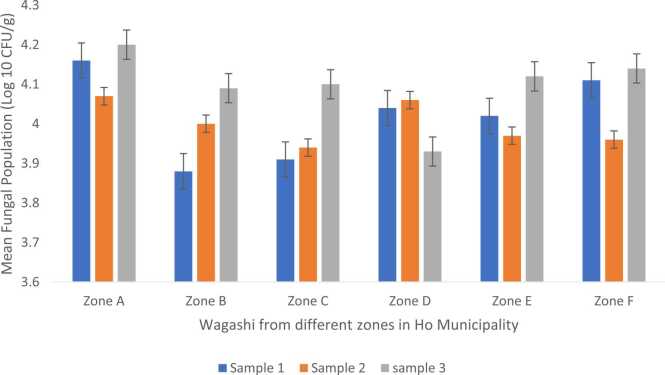
Mean fungal population in *wagashi* samples from 6 different zones in Ho municipality isolated on OGYE.

The results of the mean fungal populations on both DRBC and OGYE showed that there were significant differences (P < 0.05) observed in zones A-F. The frequent populations of the *wagashi* sample ranged from 2.36 – 4.30 log_10_ CFU/g samples (Fig. 2, Fig. 3). According to the International Commission for Microbiology Specification for Food (ICMSF, 1996) (Table 2), the quality of the *wagashi* samples from zones A – F were either marginal or potentially injurious and unfit for human consumption even by our Ghana Standard Authority Specification (>2 log_10_ CFU/g).

Abebe & Emire, (2020) reported a final population of 1.0 – 4.0 log_10_CFU/g in cheese samples from Ethiopia while Addo et al. (2021) found a fungal population of 0.91 – 1.25 log_10_ CFU/g for unprocessed cheese. In the present study, the processed *wagashi* recorded a population of 2.36 – 4.30 log_10_CFU/g from the Ho Municipality in Ghana. Catubela et al., (2019) reported a fungal population of 4.70 – 5.80 log_10_ CFU/mL for white cottage cheese in Nigeria and 4.74 – 5.71 log_10_ CFU/mL for red cottage cheese. Our present data agrees with the report of Arthur (2018) on cheese samples from Kumasi, Ghana. Hameed, [52] also recently found a fungal population of 5.99 – 6.38 log_10_ CFU (yeasts) and 0.07 – 0.66 log_10_ CFU/g (molds) in cheese in Egypt.

The cheese under investigation reported in this paper is an unripen cheese consumed in several parts of West Africa including Nigeria and, the Republic of Benin [9], [12], [65]. The Fulani people, who are pastoralists living in nomadic groups in West Africa, move their cattle from one area of grass to another where the cows give birth and produce milk, which is then processed into *wagashi* cheese. Unfortunately, milk processing in Ghana which is usually at the cottage processing level, is done under poor hygienic conditions resulting in poor microbiological quality which is underserved by the results of this paper and some others [5], [6], [17], [72] in Ghana.

The fungal species identified in *wagashi* cheese are summarized in Table 7. Potential toxigenic fungi were isolated; namely *A. flavus, A. niger, A. fumigatus, A. terreus, P. digitatum* and *Rhodotorula mucilaginosa* at various zones in the Ho Municipality.

Aflatoxins are the most occurring of all mycotoxins in both human and animal foods [91], [123], [128]. *A. flavus* strains produces aflatoxins B1, B2, G1, G2 and M1, M2. The International Agency for Research on Cancer [27], [82]; IARC, 1993) has classified AFB1 as a group 1 carcinogen and aflatoxin M1 as group 2B. Aflatoxin M1 (AFM1) is the main hydroxylated metabolite derived from aflatoxin B1 in the liver due to the cytochrome enzyme and is often detected in milk and dairy products (MDPs) [40]. When grazing, cows eat the silage, contaminated with *A. flavus* and aflatoxin AFB1 is significantly associated with AFM1 and so is possible to predict the outcome of AFM1 with knowledge of the presence of AFB1 [87]. Approximately 0.3 – 6.2% of AFB1 is converted into metabolite AFM1 and excreted in milk depending on factors such as the genetics of the animals’ seasonal variations, the milking process, and the environmental quality [23]. When dairy cows consume about 40 ug/kg AFB1 daily, as a result of feeding on contaminated silage or corn by fungi especially *Aspergillus, A. flavus, A. nomius,* or *A. parasiticus* [78], [85], consequently the milk produced could contain 0.05 ug/kg (highest level) of AFM1 (Meeting & Organization, 2002) (Joint FAO/WHO Expert Committee on Food Additives, 2002).

According to Bhaskar, [22] all around the globe consumption of unsafe food results in approximately 420,000 deaths annually and is the cause of more than 200 diseases ranging from diarrhea to cancer. The 18 *wagashi (*zones A -F) tested in this paper contained appreciable levels of AFM1 (Fig. 4 and Table 8). Samples from zones C, D, and F did not contain AFM1. On the other hand, samples from zones A, B, and E contained detectable AFM1 namely zone A (0.059±0.0002 ug/kg), B (0.02±0.01 ug/kg) E (0.02±0.001 ug/kg sample). The highest level of AFM1 was detected from zone A which was statistically different (P ≤0.05) from what was obtained in the *wagashi* from zones B and E. The variation in AFM1 levels at B and E as well as in Zones C, D, and F is an indication that;.

a) The sources of the milk for *wagashi* production may be different
b) The vendors do not produce the *wagashi* in a clean environment abiding by the standard procedure of Hazard Analysis at Critical Control Point (HACCP) and good manufacturing procedures (GMP).

Noteworthy, if the milk already contains the toxins, HACCP and GMP may not be effective strategies to curtail their occurrence. One can conjecture that the animals kept in their local dairy farms were fed with compound rations stored under poor conditions and may have favored the growth of toxigenic fungi in the genus *Aspergillus* which can in due course be contaminated with aflatoxins. The hot and humid conditions are ideal for fungal proliferation to produce mycotoxin including aflatoxin in feed and silage for the animals. It will be instinctive to design a follow-up investigation on the source and environmental quality assessment of the milk used in the *wagashi* manufacture at Ho and thus educate the nomads on the proper storage and manufacture of *wagashi* to meet international standards.

Although our results show a comparatively low concentration of AFM1 in *wagashi* samples from different localities of Ho, it must be underscored that no amount of aflatoxins is to be accepted in food (JECFA 1999). The mean AFM1 was in the range of nil to 0.06 ug/kg (0 – 60 ng/kg) in about 51.1% of the samples tested positive for AFM1 which compares favorably with what is reported in the pertinent literature. For example, [118] reported the occurrence of AFM1 (50–309 ng/kg) in 54% of samples in Iran while Ul Hassan, et al., [53] in Qatar found AFM1 in (2 – 217 ng/kg) in 85% of milk and dairy foods. Eker, Muratoglu, & Eser, [36] found 50% of samples with 19–158 ng/kg in Turkey. The mycotoxin AFM1 was recorded in Asian food as follows: China (100%); 5 – 235 ng/kg), [50]; South Korea (26%, 15 – 150 ng/kg) [64], El Salvador (South America) (92%; 5 – 485 ng/kg) Nicaragua (82%; 5 – 415 ng/kg) [110] Costa Rica (37%; 31 – 276 ng/kg) (Chavarria et al., 2015). Carvajal-Moreno, Vargas-Ortiz, Hernández-Camarillo, Ruiz-Velasco, & Rojo-Callejas, [28] found that 57% of the samples contained 1200 – 5000 ng/kg of AFM1. Lebanese samples analysed by Daou, et al., [32] recorded in 58% of the samples 19 – 1984 ng/kg. In Africa, the under-listed have been reported in milk and milk products:

1. Kenya 6800 ng/l [60]
2. Sudan (95%) contained 220 – 6800 ng/kg [37]
3. Cameroon, 15% contained 6 – 527 ng/l [122]
4. South Africa, 5 – 120 ng/l [90]
5. Nigeria, 100% contained 4 – 8450 ng/l [105]
6. Nigeria, raw cow milk AFM1 levels from nomadic cow (LOD – 31,083 ng/L) [8]
7. Egypt, AFM1 levels in raw milk 23 – 73 ng/kg [45]
8. Egypt 8.57 – 2120 ng/kg [58], [90]

Aflatoxin exposure early in life has been associated with impaired growth especially stunting (Gong et al., 2002). Early exposure to aflatoxins is a potential risk for synergistic interaction with other toxins [13]. It is recorded that greater quantities of AFM1 were detected in other parts of the world than in Africa. In the global context, AFM1 locals in Ghanaian milk and cheese products like *wagashi* are moderate [66], [72]. Flores-Flores, Lizarraga, de Cerain, & González-Peñas, [42] reviewed the presence of AFM1 in cow milk and products from various parts of the world. Out of the 22,189 milk samples analyzed, 9.8% of them (2190 samples) exceeded the maximum AFM1 content established by the EU. Exactly 1709 came from Asia, 253 from Africa, 119 from Europe, and 109 from America. Several factors such as geographical region, season, type and quality of feed, storage, and items, and processing methods are conditions that are responsible for the variability of AFM1 in milk and dairy products [120]. In this paper, even among different zones A – F in the municipality of Ho, there were marked variations in the AFM1 detected in *wagashi* in consonance with the microprint of Gizachew, Szonyi, Tegegne, Hanson, & Grace, [46] and [120].

According to Kuiper-Goodman, [73], risk assessment/estimations are modeled to predict the adverse health implications of mycotoxin exposure and guide food regulators to set thresholds for toxins in foods. The margin of Exposure (MOE) obtained in these present results implies a high risk for infants, children, adolescents, and adults in the Ho Municipality, as all calculated MOE values were less than 100,000. The European Food Safety Authority (EFSA, 2007) states categorically that an MOE of 10,000 or more indicates a situation of low public health concern. As all the MOEs calculated were far below 10,000, it suggests that AFM1 exposure in all age categories across the board poses a high public health risk. As an inference, the smaller the MOE, the larger the potential risk posed by exposure to dietary AFM1 [103].

There were other fungi isolated from the *wagashi* which are of human health importance [81]. For example, *Rhodotorula mucilaginosa* is a yeast commensal with similar characteristics to the *Cryptococcaceae* and it causes fungemia attributed to medical devices such as bronchoscopes and central venous catheter (CVC) [54]. This candidemia presents as salmon-pink colonies in blood cultures [31] and forms a biofilm of clinical and environmental importance [95]. This fungus has high resistance to antifungal therapy MCFG (Microfungi), azoles, and echinocandins [31].

*Aspergillus terreus* although sparsely encountered in the *wagashi* also presents a health hazard. It produces a large number of secondary metabolites and mycotoxins. *A. terreus* produces mycotoxins such as citreoviridin [43], patulin, citrinin, teretonin and gliotoxin [11], [14], [117], [124]. *A. terreus* produces a plethora of several anticancer bioactive compounds such as statin group of polyketides lovastatin that are routinely used to treat lipid disorders including hypercholesterolemia and cardiovascular diseases, compactin, pravastatin, mevastatin, simovastin; it is also reported as producing a non-neolignan compound, asperjinon along with twelve other known compounds [76]. In addition, *A. terreus* is a plant pathogen decaying over 120 million tons of food grain [80] and could have been part of the phylloplane fungi that contaminate the silage used as fodder for the considered foraging.

*Aspergillus fumigatus* was one of the predominant fungal contaminants of the *wagashi* (Table 3, Table 4). *A. fumigatus* produces a mycotoxin called fumagillin [51], [113]. Fumagillin has activity on its target, the methionine peptidase type 2 (metAP2 enzyme). The same fungus also produces several mycotoxins such as gliotoxin and pseudoritin [51]. Fumagillin can inhibit the function of neutrophils in blood inducing cell death in erythrocytes and also plays a role in the damage of epithelial cells which opens the way for fungal evasion [44], [51]. Therefore, the presence of *A. fumigatus* in cheese fermented from cow milk (*wagashi*) in Ghana cannot be taken lightly and discriminated against as it has serious health implications and toxic effects on human functions such as metabolism [51], [70], [113]. There are records in the pertinent literature that *A. fumigatus* also produces other mycotoxins such as fumitremorgans, verruculogen, and gliotoxin and can cause aspergillosis in both humans and animals [114], pulmonary aspergillosis (lung), aspergilloma (fungal balls), skin and nail infection as well as eye and ear infections [111]. These findings open a new direction of study to ascertain the presence of fumagillin and other ancillary mycotoxins formed by *A. fumigatus* in the cheese *wagashi* stored and eaten in Ghana.

Although *A. nige*r is not known to produce aflatoxins or fumagillin, it possesses the ability to produce other toxins such as ochratoxin A, malformin and nigerone [108].

*Fusarium verticillioides* (=*F. moniliforme*) also frequently isolated from *wagashi* cheese in this study produces fumonisin, which has a neurotoxic effect in animals and is associated with esophageal cancer in sub-Saharan Africa [20], [29]. These fungal species were presumably part of the phylloplane fungi of the silage used in feeding the cows or might have contaminated the cheeses during preparation. Another interesting observation of pathological importance was the presence and isolation of *F. oxysporum* from most of the zones (Table 3, Table 4, Table 5). *F. oxysporium* is a well-known plant pathogen that causes severe damage to many crops, both in the field and during post-harvest storage (Mondani et al., 2021; De Lamo and Takken, 2020). *F. oxysporum* is famous for its ability to cause wilt, root, and fruit rot in many plant species including agricultural important crops (Dean et al., 2012). This fungus ranks among the 10 most devastating fungal threats to agricultural productivity (Dean et al., 2012) and wilts are a major threat to agricultural productivity (Fisher et al., 2012). It is conjectured that presumably brought them into contact with these species through the silage which in turn emerged in the cheese prepared from their milk.

## 5 Conclusion and recommendations

Data from this study suggest that the fungal populations recorded in the *wagashi* samples from six zones in the Ho Municipality were high (2.36 – 4.30 log_10_ CFU/g sample) and potentially injurious and unfit for human consumption according to the ICMSF (1998) specifications. The fungal profile showed the presence of thirteen (13) fungal species belonging to eight (8) genera (*Aspergillus, Fusarium, Mucor, Penicillium, Rhizopus, Rhodotorula, Trichoderma* and other yeasts). Some toxigenic *Aspergillus* species (*A. flavus, A. fumigatus, A. niger, A. terreus*) and *Fusarium* (*F. oxysporium, F. verticillioides*) and *Penicillium* (*P. digitatum*) not excepting *Rhodotorula mucilaginosa* were isolated.

Aflatoxin M1 (AFM1) concentrations in *wagashi* ranging from 0.000 (N.D) ± 0.00 to 0.0592 ± 0.002 ug/kg were detected at zones A, B, E, and F (60%) but none at zones C, D (40%). It was established that the consumption of *wagashi* could pose adverse health threats to all age categories (infants, toddlers, children, adolescents, and adults) in the zones samples as exposure and risk assessment showed a possible adverse health and cancer risk. The Margin of Exposure (MOE) obtained in this present work was less than 100,000 implying a high public health risk according to the European Food Safety Authority, EFSA (2007).

## Ethics approval and consent to participate

Not applicable.

## Author contribution

NKK, VSG, and GTO: Conceived and designed the experiments; performed the experiments; analyzed and interpreted the data; contributed reagents, materials, analysis tools, or data; NKK, VSG, GTO: wrote the paper. VSG, LOA, VOK-B, and MW-K: performed experiments. VSG, GTO, and NKK: Analyzed and interpreted the data.

## Funding

No funding was received for this research.

## CRediT authorship contribution statement

**Ansah Leslie Owusu:** Data curation, Formal analysis, Methodology, Validation. **Wiafe-Kwagyan Michael:** Data curation, Methodology, Resources, Writing – review & editing. **Gillette Valentina Sylvia:** Data curation, Investigation, Resources, Writing – original draft, Writing – review & editing. **kortei nii korley:** Conceptualization, Formal analysis, Investigation, Methodology, Resources, Validation, Writing – original draft, Writing – review & editing. **Odamtten George Tawia:** Conceptualization, Formal analysis, Methodology, Supervision, Visualization, Writing – review & editing. **Kyei-Baffour Vincent:** Formal analysis, Methodology, Validation.

## Declaration of Competing Interest

The authors declare that they have no known competing financial interests or personal relationships that could have appeared to influence the work reported in this paper.

## Data Availability

Data will be made available on request.
